# Taper wear in total joint arthroplasty can be reliably assessed with various coordinate measuring systems

**DOI:** 10.1038/s41598-025-96760-7

**Published:** 2025-04-06

**Authors:** Therese Bormann, Ulrike Mueller, Robert Sonntag, Stefan Schroeder, J. Philippe Kretzer

**Affiliations:** Department of Orthopaedics, Research Center of Biomechanics and Implant Technology, Heidelberg University Hospital, Schlierbacher Landstrasse 118a, 06118 Heidelberg, Germany

**Keywords:** Trunnion, Total hip arthroplasty, Taper corrosion, Modular implant, Coordinate measuring machine, Preclinical research, Biomaterials, Techniques and instrumentation

## Abstract

In total joint arthroplasty, wear and corrosion at modular taper junctions is an issue with clinical implications, as ions and wear debris can lead to adverse tissue reactions. The quantification of the generated wear is, therefore, an important measure to judge the performance of such modular junctions. This applies to pre-clinical in vitro investigations as well as to retrospective investigations of retrieved implants. The volume of the worn material can be determined with coordinate measuring machines (CMMs), which can generally be classified as tactile and optical systems. The study aims on the comparison of a tactile with two optical CMM systems for the determination of taper wear. To do so, four taper samples—three trunnions and one bore taper—with different amounts of known volumetric wear (range 1.5 mm^3^ to 8.3 mm^3^) were fabricated. Wear volume, linear deviation and taper angle were determined with the different CMM systems. The tactile system yielded the highest deviation from the gravimetric reference values of about 0.3 mm^3^, while the optical systems exhibited deviations of about 0.1 mm^3^ and 0.2 mm^3^. Clinically relevant taper wear, however, is well measurable with all investigated systems.

## Introduction

In total joint arthroplasty, modularity is an important tool to adjust the endoprosthesis to the patient specific situation. Also, modularity allows the replacement of individual implant parts in case of a revision operation, without the need for the exchange of well-integrated components. Modular connections in joint endoprostheses are often realized as taper connections. The primary artificial hip joint possesses a taper connection between the femoral head and the hip stem. After concerns about its resistance towards fretting-corrosion arose^1^, this taper connection has been extensively studied for example with respect to wear mechanisms, the contact micromechanics, geometrical taper parameters, influence of surface topography etc^2–7^. There are, however, numerous other taper junctions available in hip, knee and shoulder arthroplasty. Especially in revision systems, manufacturers provide modular systems to bridge large bone defects and/or to account for specific anatomic conditions. An inherent risk of modular junctions is the degradation due to wear and corrosive processes. As ions and wear particles are released, these can lead to metal related clinical implications such as adverse local tissue reactions, including for example pseudotumor formation, tissue necrosis and periprosthetic osteolysis^8^. Furthermore, taper wear may also play a role in the rare event of taper junction breakage^9–11^. The quantification of occurring material wear is, therefore, an important measure to judge the performance of the modular connections. Wear processes at taper connections in vivo are, however, complex, as the occurring tribo-corrosive processes depend on a variety of parameters. From the technical point of view, these are for example the used material involving also the fabrication parameters, the constitution of the paired surfaces (composition and topography) or contact geometry of the two surfaces^12–14^. On the other hand, patient specific parameters like individual loading regimes or the constitution of the synovial fluid (electrolyte) also play a role in the tribo-system^15–17^, which makes the in vitro simulation of the in vivo situation challenging. Retrieval analysis offers the advantage to evaluate the in vivo performance of tapered junctions retrospectively but generally lacks the possibility of a comparison to the component in an unworn state. Quantification of wear volume from retrieved implants is nevertheless accomplished by mapping taper surfaces with roundness measurement machines (RMM) or coordinate measuring machines (CMM)^18,19^. The ‘original’ taper surface is generated by extrapolating the taper geometry from unworn regions of the part, which are often characterized by the presence of as-manufactured surface regions. The wear volume is determined from the difference between the constructed ‘original’ and the actual taper surface^20^. In in vitro investigations, volumetric wear can be measured by other, more straightforward techniques like for example gravimetry or ion analysis^15^. The quantification of wear by geometric measurements offers, however, the advantage of determining local wear patterns, while gravimetry or ion analysis yields an overall value for the amount of worn off material.

Tactile RMM or CMM systems for geometric measurements involve styli, whose tips typically have dimensions between 2 μm (RMMs) and 5 mm (CMMs). The tip geometry influences the measurement result since especially styli with relatively large and round tips as used in CMM systems act as a morphological filter. This means that small-scale roughness features, such as for example the surface profiles of grooved stem-tapers, are not completely captured within the measurement, which can lead to measurement errors. Non-contact, optical CMMs, on the other hand, yield data with a much higher point density than tactile CMM systems and are considerably faster. The optical systems, therefore, can also resolve features of the surface topography which yields a much more detailed picture of the measured part. Optical systems, however, may be prone to measurement errors caused by reflective surfaces or by alterations in optical properties within the measured part. Also, measuring of internal surfaces, such as in bores or head tapers, is limited. The study aims on determining whether optical coordinate measurement systems are comparable to a tactile system if wear on conical surfaces is quantified. We, therefore, measured artificially generated taper wear with three different coordinate measurement machines, a tactile system and two optical systems. The optical systems are based on two different techniques, namely focus variation and chromatically encoded confocal measurement.

Research questions are: (1) How accurate is the determination of the volumetric wear of taper surfaces with different coordinate-based measurement techniques? (2) Are optical systems comparable to a tactile system?

## Materials and methods

### Test samples

The test samples were three 12/14 trunnions fabricated from Ti6Al4V and one 12/14 bore taper from CoCrMo alloy as often used in total hip arthroplasty at the head-stem modular junction. Taper length was 18 mm for the trunnions and 22 mm for the bore taper.

The trunnions were manufactured with a grooved surface topography, while the bore taper had a smooth surface profile (see Table 1). Surface roughness was measured with a tactile profilometer (M2, Mahr, Göttingen, Germany). The taper angle was determined by measuring the taper geometry with a tactile coordinate measuring machine (MS222, Mahr, Göttingen, Germany). After investigating the initial roughness and taper angles, taper wear was artificially generated at the trunnions by careful grinding with SiC-paper, while the trunnion was rotating in a turning machine. For the bore taper, a rotating grinding device was used (FBS 240/E, Proxxon, Niersbach, Germany). The created wear scar stretched out over a width of about 3–5 mm in taper axis direction. Care was taken to leave unworn surface regions above and underneath of the wear scar in order to allow the creation of a referencing cone from unworn surface areas. The wear pattern chosen corresponds to the engagement pattern of Type I according to ASTM F3129-16^20^. Taper wear was generated to represent clinically relevant material loss from head-stem taper junctions, which range from about 1 mm^3^ to more than 20 mm^3^^21–23^. After generating wear, all samples were cleaned in an ultrasonic bath with a 1% detergent solution (EM-080 universal concentrate, EMAG Technologies, Mörfelden-Walldorf, Germany) for 15 min and subsequently rinsed and dried. Wear volume was gravimetrically determined with a high precision balance (ME 235 S-OCE, Sartorius AG, Göttingen, Germany) under the assumption of a density of 4.43 g/cm^3^ and 8.3 g/cm^3^ for Ti6Al4V and CoCrMo samples, respectively. The measurement error of weighing was in the range of 3 * 10^−5^ mg and is considered negligible.

**Table 1:** Material, initial roughness values R_a_, R_z_ and R_sm_, initial taper angle and generated wear volume of the samples used.

Sample		Trunnion 1	Trunnion 2	Trunnion 3	Bore taper
Material		Ti6Al4V	Ti6Al4V	Ti6Al4V	CoCrMo
R_a_ / µm		0.96 ± 0.04	0.95 ± 0.02	0.95 ± 0.03	0.32 ± 0.02
R_z_ / µm		4.64 ± 0.22	4.63 ± 0.18	4.63 ± 0.16	1.82 ± 0.34
R_sm_ / µm		96.07 ± 0.42	96.57 ± 1.43	95.83 ± 0.27	28.47 ± 0.30
Taper angle / °		5.650	5.649	5.657	5.777
Wear volume / mm^2^		1.40	5.24	8.21	4.13

### Determination of wear volume by geometric measurements.

#### Tactile coordinate measurement machine

The wear volume was geometrically determined with the tactile coordinate measurement machine MS222 (Mahr, Göttingen, Germany), which has a measurement accuracy of 3 μm. A stylus of 30 mm length with a 3 mm diameter ruby tip was used. The taper was measured at the circumferential surface along 15 mm of its length, starting two mm underneath of the frontal face. Point spacing was 6° in circumferential direction and 0.5 mm in the direction of the taper axis resulting in 1860 measuring points per specimen. It has been shown that with tactile CMMs, taper wear can be assessed with data point spacings up to 1 mm × 1 mm^24^. Own preliminary investigations also indicated that increasing the point density above the applied parameters does not increase the accuracy of the resulting wear volume (but does considerably increase the measurement time). Measured point clouds were further processed in Imageware™ (v.12.2, UGS Corporation, Plano, TX, USA) and Matlab (R2019a, The MathWorks, Inc., Natick, MA, USA). First, the unworn surface regions were identified by the difference between the measured point cloud and a cone fit from the point cloud in combination with the knowledge about the location of the wear scar. The worn regions were than manually removed from the point cloud. Subsequently, the reference cone was generated from the unworn surface regions in Imageware™ by approximating the best fit non-uniform rational basis spline (NURBS) cone for the data points with minimum error. Subsequently, the normal deviation between each measuring point and the referencing cone was determined. Afterwards, the wear volume for each individual measuring point was calculated as product of the normal deviation and the surface area that is represented by the respective point. The total wear volume was determined by summing up the individual results for each measuring point. Each sample was measured and analysed three times, i.e. the measurement error of the determined wear volume refers to the standard deviation of the three results for each sample.

#### Optical CMM system I

The test samples were measured with the µCMMReal3D (Bruker-Alicona, Graz, Austria), which has a measurement accuracy of about 0.8 μm. An 800 A objective was used. 1.1 million measurement points were used for trunnion samples and 2.2 million measurement points were used for the bore taper.

Data analysis was done with GOM Inspect Suite 2020 (Carl Zeiss GOM Metrology, Braunschweig, Germany). The data analysis protocol was established with respect to the data analysis procedure used for the tactile CMM: First, a mesh was created from the point cloud. From the mesh, the reference cone was generated from the unworn surface regions by only using the areas above and underneath of the wear scar for cone fitting. Due to the high number of measured data points, the wear scar could be identified visually from the mesh, as the worn regions exhibited an altered surface roughness than the original surface. Subsequently, the mean value of the deviations (D_mean_) between referencing cone and mesh in the worn region of the sample was determined. The wear volume was calculated as product of D_mean_ and the surface of the region of material wear from the referencing cone. Each sample was measured once because of the lengthy measurement time. The wear volume was determined three times for each sample. The measurement error of the wear volume refers to the standard deviation of three individual data analyses for each sample.

#### Optical CMM system II

The second optical CMM system was the OmniLux 4 AB (Redlux Ltd, Romsey, United Kingdom). The measurement accuracy of the system is 0.15 μm. A replica from the bore taper was generated by a validated molding technique^25^ prior to the measurement because the sensor head of the system is too big to be inserted in a 12/14 bore taper. The test samples were measured with about 250,000 measuring points. The OmniLux CMM provides a software that allows to directly determine the wear volume from tapers or spheres, based on the difference of the measured data and a reference surface fitted by linear least-squares fit to unworn sample areas^26^. The unworn regions were visually determined from the sensor intensity signal, which yields a vivid representation of the measured part. Measurement and wear volume determination was done twice for each specimen. The measurement error of the wear volume was defined as the maximal difference between the individual results.

## Results

Gravimetrically, the wear volume accounted to 1.40 mm^3^, 5.24 mm^3^ and 8.21 mm^3^ for the trunnion samples and to 4.13 mm^3^ for the bore taper. The mean wear volume determined with the tactile CMM was 1.82 mm^3^ ± 0.01 mm^3^, 5.43 mm^3^ ± 0.12 mm^3^ and 8.40 mm^3^ ± 0.04 mm^3^ for the trunnions 1, 2 and 3, respectively, and to 3.71 mm^3^ ± 0.02 mm^3^ for the bore taper. The measurements with the optical CMM I resulted in mean wear volumes of 1.45 mm^3^ ± 0.08 mm^3^, 5.25 mm^3^ ± 0.21 mm^3^ and 8.42 mm^3^ ± 0.1 mm^3^ for the trunnions and to 4.0 mm^3^ ± 0.07 mm^3^ for the bore taper. The optical CMM II yielded mean wear volumes of 1.2 mm^3^ ± 0.1 mm^3^, 5.0 mm^3^ ± 0.1 mm^3^ and 8.0 mm^3^ ± 0.1 mm^3^ for the trunnions and 4.1 mm^3^ ± 0.1 mm^3^ for the bore taper. The tactile CMM showed a mean deviation of 0.30 mm^3^ ± 0.36 mm^3^ from the gravimetric reference values. The Optical CMM I showed the smallest mean deviation of 0.10 mm^3^ ± 0.09 mm^3^, i.e. the CMM I almost exactly reproduced the reference values. The optical CMM II exhibited a mean deviation of 0.20 mm^3^ ± 0.09 mm^3^.

Measurements with the tactile CMM and the optical CMM I overestimated the wear volume in case of the trunnions and underestimated the wear volume in case of the bore taper. The optical CMM II, on the other hand, systematically underestimated the wear volume of about 0.2 mm^3^.

**Fig. 1:**
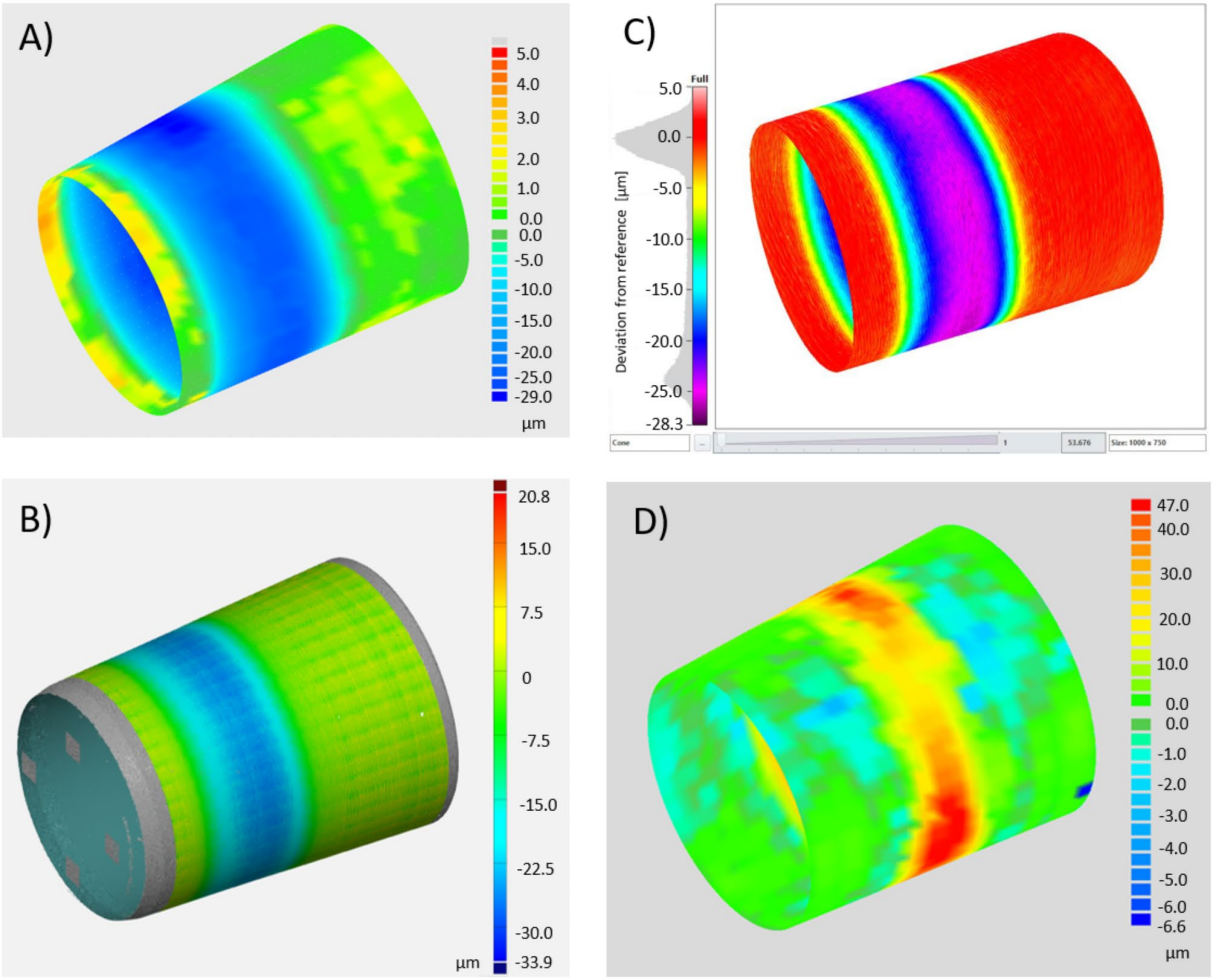
The deviation from the referencing geometry (color coded) clearly shows the area of material abrasion. (**A**) Trunnion 2, tactile system; (**B**) Trunnion 2, optical CMM I; (**C**) Trunnion 2, optical CMM II; (**D**) Bore taper, tactile system.

With the tactile CMM, the maximum linear deviation from the referencing cone surface was about 12 μm, 28 μm and 39 μm for the trunnion samples and 47 μm for the bore taper. The optical CMM 1 yielded values of 19 μm, 34 μm, 45 μm and 64 μm, while the optical CMM II resulted in values of 11 μm, 28 μm, 40 μm and 98 μm. In the plots of the linear deviation from the referencing surface (Fig. 1) the region of material wear is clearly represented.

The mean linear deviation was about 8 μm, 18 μm and 25 μm for the trunnion samples and 20 μm for the bore taper with the tactile CMM. The optical CMM I yielded values of 4 μm, 15 μm and 23 μm for the trunnions and 16 μm for the bore taper. From optical CMM II, mean linear deviations were not determined.

In addition to the wear volume, also the taper angles were determined from the data sets of the samples with generated taper wear. The angles were determined from the referencing cones, which were created for determination of the wear volume. With the tactile CMM, taper angles were 5.654° ± 0.001°, 5.667° ± 0.005° and 5.659° ± 0.002° for the trunnions 1, 2 and 3, respectively and 5.772° ± 0.001° for the bore taper. CMM I yielded taper angles of 5.658°± 0.001°, 5.667° ± 0.014° and 5.661° ± 0.004° for the trunnions and 5.871° ± 0.001° for the bore taper. CMM II yielded taper angles of 5.646°, 5.649° and 5.643° for the trunnions and 5.752° for the bore taper. The mean deviation from the initial taper angles was, therefore 0.01° ± 0.009° for the tactile CMM, 0.031° ± 0.042° for the optical CMM I and 0.011° ± 0.011° for the optical CMM II (cp. Table 2).

**Table 2:** Wear volume, maximum linear deviation, mean linear deviation and taper angle of the four taper samples, determined with different CMMs. Also, the mean deviation from the referencing values (taper wear) and the initial taper angles is shown.

	Trunnion 1	Trunnion 2	Trunnion 3	Bore taper	Deviation from reference
Wear volume / mm^3^					
Gravimetry (reference)	1.40	5.24	8.21	4.13	–
Tactile CMM	1.82 ± 0.01	5.43 ± 0.12	8.40 ± 0.04	3.71 ± 0.02	0.30 ± 0.36
Optical CMM I	1.45 ± 0.08	5.25 ± 0.21	8.42 ± 0.1	4.0 ± 0.07	0.1 ± 0.14
Optical CMM II	1.2 ± 0.1	5.0 ± 0.1	8.0 ± 0.1	4.1 ± 0.1	0.2 ± 0.09
Maximum linear deviation / µm					
Tactile CMM	11.7 ± 0.4	28.2 ± 0.2	38.9 ± 0.4	47.4 ± 0.3	–
Optical CMM I	19.0 ± 0.1	33.6 ± 0.5	44.9 ± 0.5	63.7 ± 0.2	–
Optical CMM II	10.9	28.3	40.4	98.4	–
Mean linear deviation / µm					
Tactile CMM	7.7 ± 0.1	17.5 ± 1.0	24.7 ± 0.1	20.1 ± 0.2	–
Optical CMM I	4.1 ± 0.4	14.9 ± 0.7	23.1 ± 0.7	16.3 ± 1.8	–
Taper angle / °					
Initial	5.650	5.649	5.657	5.777	–
Tactile CMM	5.654 ± 0.001	5.667 ± 0.005	5.659 ± 0.002	5.772 ± 0.001	0.01 ± 0.009
Optical CMM I	5.658 ± 0.001	5.667 ± 0.014	5.661 ± 0.004	5.871 ± 0.001	0.031 ± 0.042
Optical CMM II	5.646	5.649	5.643	5.752	0.011 ± 0.011

## Discussion

Generally, all investigated methods yielded results that were in reasonable agreement with the reference values. The biggest individual deviation from the reference value was 0.42 mm^3^ for the tactile CMM. In general, the tactile CMM exhibited higher deviations from the reference values, while the optical CMM I yielded the smallest mean deviation. For tactile CMM wear measurements on taper surfaces, an accuracy of 0.2 mm^3^ was reported in a former study that applied a CMM with an accuracy of 0.8 μm^27^, i.e. a tactile CMM with higher accuracy might yield more accurate results of volumetric wear.

A limitation of the study is the ideal arrangement of the worn areas, which left rather large unworn taper portions above and underneath of the wear scar and allowed a good estimation of the referencing surface with respect to the original taper. This type of engagement pattern is defined as Type I in ASTM F3129-16^20^. Such unworn regions above and underneath of a wear scar appear in head tapers in case of short and completely inserted stem tapers. For stem tapers, however, unworn areas at the distal taper end only are more likely (Type II engagement pattern according to ASTM F3129-16)^20^. Such a scenario with little and/or localized unworn taper surface regions can lower the accuracy of the geometry of the referencing surface with respect to the original taper. As the geometry of the original taper surface should be estimated as good as possible, the measurement precision of the investigated technique may be lowered in case of ‘real’ (retrieved) stem taper samples.

**Fig. 2:**
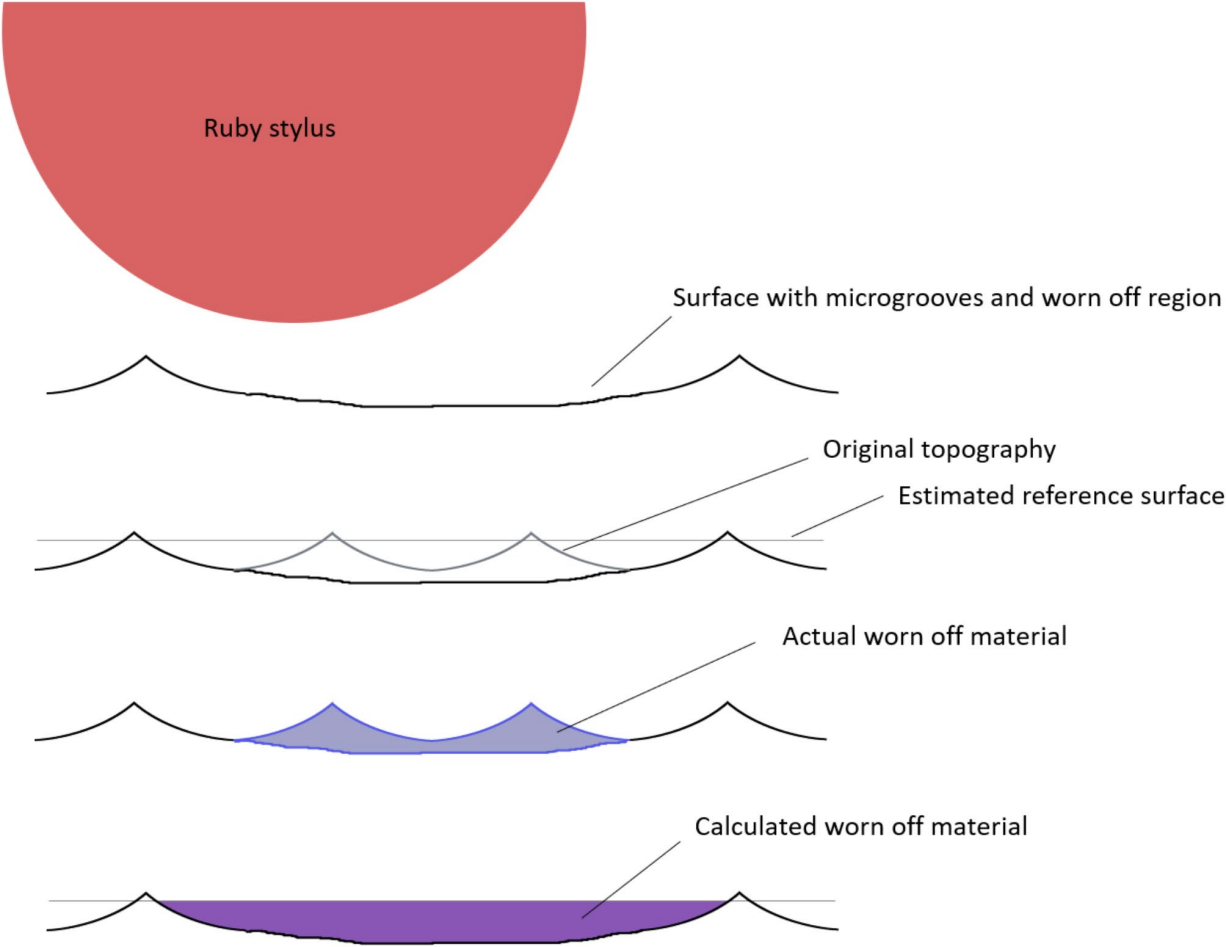
Schematic representation of the overestimation of volumetric wear due to a micro-grooved surface topography and a spherical stylus. Please note that microgrooves and stylus are not represented true to scale.

With tactile CMMs, point spacings between 0.1 and 1 mm have been used^20^. Such rather large point spacings do not represent the surface with topographical features in the µm-range. In addition, the stylus with a round tip of several millimeters acts as a morphological filter, which removes certain features of the roughness^28^, like for example the micro-grooved topography of stem tapers. This “morphological filtering” can result in a systematic over- or underestimation of the wear volume, depending on the surface topography. If the taper has a micro-grooved topography, the spherical stylus, which is large in comparison to the topographical features, leads to an overestimation of wear volume. This is because the top surface of the created referencing face is in the range of the contact zone between stylus and profile tops, since the bottom can not be reached with the stylus, and the void volume in between the profile tops is not accounted for (see Fig. 2). The overestimation becomes larger with the roughness parameters of the surface. For this reason, roundness measuring machines with much smaller diameter diamond styli have been suggested to determine the wear volume of micro-grooved stem tapers^18^. In the data analysis procedures of roundness measuring machines, however, specific and individually tailored protocols were implemented to take the void volume of the micro grooves into account^18,29^, which may be difficult and time consuming when it comes to the investigation of large retrieval collections. On smooth surfaces without micro grooves, on the other hand, the spherical stylus leads to an underestimation of the wear volume caused by the removal of small scale roughness features^20^. The differences in surface topography are possibly reflected in the overestimation of taper wear in case of trunnions and the underestimation in case of the bore taper for the tactile CMM. The optical systems, in contrast, do resolve the micro-grooved surface topography, but the topography is still not considered in the calculation of the wear volume. This is because the referencing cone is constructed from fitting a plain geometric surface (or corpus) into the measured point cloud (or the meshed surface), which also ignores the grooved topography. The user has to be aware that the resulting wear volume depends on the cladding conditions of the fitting strategy.

The used trunnion samples exhibited roughness features with an amplitude of about 5 μm and a wavelength of about 100 μm. A taper surface is considered micro-grooved if the amplitude is > 4 μm and the wavelength > 100 μm^30^. The calculated wear volume of the used test samples, however, was in good agreement with the actual wear volume. As the surface topography of the trunnions was on the lower end of what is considered micro-grooved, the error due to a micro-grooved surface can be expected to be larger in case of trunnions that exhibit a higher surface roughness. At hip stem tapers (trunnions), the surface roughness varies broadly with amplitudes between about 3 and 50 μm, while at head tapers the surface normally does not comprise a grooved topography and can be considered as smooth^31^.

In tactile CMM systems, each measuring point is set at an exactly defined location within the taper surface. This means that for each data point the referencing portion of the surface can exactly be determined and the wear volume is determined by integrating the individual volume elements^24^. The optical CMM I, on the other hand, yielded point clouds with point amounts of about three orders of magnitude higher than the amount of data points of the tactile system. Here, the data points were rather randomly distributed, which makes it impossible to determine the portion of the taper surface for each individual measuring point. The wear volume was, therefore, calculated as product from the area of the wear region and the arithmetic mean of the deviations between the measured data and the referencing cone surface in this respective region. This approach worked out very well, while another approach, in which the wear volume was calculated as difference between two three-dimensional tapers (referencing cone, i.e. closed referencing surface, and measured taper including worn regions), yielded results with deviations > 0.5 mm^3^ from the gravimetric wear values. Errors in determination of the wear volume can, therefore, be introduced in the course of data processing. For the tactile CMM and CMM I, data analysis protocols were established to determine the volumetric material loss from the measured point clouds. For the CMM II, data analysis with respect to volumetric material loss is already implemented in the software that comes with the system. Therefore, using the CMM II was the most straightforward and quick method to determine the wear volume of taper surfaces.

Taper wear has also been evaluated using the parameter linear deviation between referencing face and actual taper surface^27,32^. The mean linear deviations from the referencing face are less sensitive towards individual measurement values than maximum linear deviations and can be considered a viable alternative to the volumetric measurements. The mean linear deviations showed good agreement between the tactile system and the CMM I. For CMM II, no mean linear deviations were derived. With respect to maximum linear deviations, the measured values were in good agreement for all three CMMs in case of the trunnions. The optical CMM I yielded the highest values for maximum linear deviations, which might be attributed to the high point cloud density. The maximum deviations derived from the three the individual systems are, however, much larger for the bore taper. Here the difference between the three CMMs was as high as about 50 μm, with CMM II yielding a much larger value than the other two systems. When it comes to bore tapers, this difference in maximum linear deviation might be attributed to the different scanning strategies. For a stylus-based system, the stylus can be inserted into the bore as long as it is long enough, i.e., measurement accuracy is not altered in comparison to the measurement of trunnions, as simply the direction of the movement has to be inverted. The CMM I, on the other hand, orients the bore axis in parallel to the light source, i.e. the tapered surface is scanned almost vertically, which limits the possible measurement depth. Also, the resolution may be altered in comparison to outer tapers (trunnions), which allow a perpendicular orientation to the light source and so an optimal illumination of the sample. CMM II uses a different approach, here the surface is replicated to allow the investigation of the outer surface, as done in stem tapers. This approach has been shown to introduce variations in measured wear volume of up to ± 0.16 mm^3^^25^, i.e. the maximum deviation might be slightly higher than calculated from the conducted measurements. As wear volume could, however, be reliably assessed, the mentioned drawbacks did not noteworthy influence the measurement results. The optical CMM I, however, showed a rather high deviation of the measured head taper angle of about 0.09°, while the agreement for the stem taper angles was comparable to the other systems. The high head taper angle variation with CMM I may be a result of variations in sample illumination depending on the height position within the measurement in the bore taper.

With respect to in vivo taper wear from hip stem tapers, values for average volumetric wear at retrieved stem tapers were reported to lie in a range about between 0.1 and 0.3 mm^3^ in case of well-functioning stem tapers^18,19,32^. In contrast, mean volumetric material loss of 1.3 mm^3^, which can expand to values of more than 20 mm^3^, was found in severely corroded stem tapers^21^. In CoCrMo heads, taper wear is significantly higher than at the stem tapers. Retrieval studies reported a range of average volumetric head taper wear between 1 and 6.2 mm^3^ ^18,21–24,32−34^. It remains, however, difficult to determine a threshold for taper wear that leads to clinical complications^35^. In MoM and MoP retrieval collections, which were revised mainly because of metal related complications, head taper material loss ranged between 1 and 1.6 mm^3^ (median values), respectively^22,23^. With well-functioning taper connections, however, head taper material loss can also be very low. A study of Langton et al. reported a median of 0.17 mm^3^ material loss for optimal working Exeter V40 head tapers^36^. Taper wear of well working taper connections, therefore, may be hard to be determined with all CMM systems tested in this study, since reported wear volumes are often in the range of the accuracy of the CMM systems. Clinically critical taper wear from head and stem tapers can be determined reliably with all investigated systems.

## Data Availability

The datasets generated during and/or analysed during the current study are available from the corresponding author on reasonable request.

## References

[1] Morlock, M., Bünte, D., Gührs, J. & Bishop, N. Corrosion of the head-stem taper junction—Are we on the verge of an epidemic? Review article. *HSS J.* **13**, 42–49 (2017). 28167873 10.1007/s11420-016-9526-4 PMC5264576

[2] Osman, K., Panagiotidou, A. P., Khan, M., Blunn, G. & Haddad, F. S. Corrosion at the head-neck interface of current designs of modular femoral components. *Bone Joint J.* **98-B**, 579–584 (2016). 27143725 10.1302/0301-620X.98B5.35592

[3] Mistry, J. B. et al. Trunnionosis in total hip arthroplasty: A review. *J. Orthop. Traumatol.* **17**, 1–6 (2016). 26868420 10.1007/s10195-016-0391-1 PMC4805640

[4] Balachandran, S. et al. Atomic scale origin of metal ion release from hip implant taper junctions. *Adv. Sci.* **7**, 1903008 (2020). 10.1002/advs.201903008 PMC7055581 32154080

[5] Feyzi, M., Fallahnezhad, K., Taylor, M. & Hashemi, R. A review on the finite element simulation of fretting wear and corrosion in the taper junction of hip replacement implants. *Comput. Biol. Med.* **130**, 104196 (2021). 33516962 10.1016/j.compbiomed.2020.104196

[6] Mueller, U., Bormann, T., Schroeder, S., Renkawitz, T. & Kretzer, J. P. Taper corrosion in total hip arthroplasty—How to assess and which design features are crucial? *J. Mech. Behav. Biomed. Mater.* **133**, 105307 (2022). 35688037 10.1016/j.jmbbm.2022.105307

[7] Pourzal, R. et al. Does surface topography play a role in taper damage in Head-neck modular junctions? *Clin. Orthop. Relat. Res.* **474**, 2232–2242 (2016). 27339123 10.1007/s11999-016-4933-x PMC5014821

[8] Hall, D. J., Pourzal, R. & Jacobs, J. J. What surgeons need to know about adverse local tissue reaction in total hip arthroplasty. *J. Arthroplasty* **35**, S55–S59 (2020). 32005621 10.1016/j.arth.2020.01.016 PMC7239747

[9] Butler Ransohoff, C. et al. The different failure modes of the connecting elements of the modular hip arthroplasty revision stem revitan. *J. Mech. Behav. Biomed. Mater.* **123**, 104778 (2021). 34416537 10.1016/j.jmbbm.2021.104778

[10] Norman, P., Iyengar, S., Svensson, I. & Flivik, G. Fatigue fracture in dual modular revision total hip arthroplasty stems: Failure analysis and computed tomography diagnostics in two cases. *J. Arthroplasty* **29**, 850–855 (2014). 24120507 10.1016/j.arth.2013.09.008

[11] Krueger, D. R., Guenther, K. P., Deml, M. C. & Perka, C. Mechanical failure of 113 uncemented modular revision femoral components. *Bone Joint J.* **102-B**, 573–579 (2020). 32349597 10.1302/0301-620X.102B5.BJJ-2019-1333.R2

[12] McCarthy, S. M. et al. Has wrought cobalt-chromium-molybdenum alloy changed for the worse over time? *J. Arthroplasty* **38**, S280–S284 (2023). 37028774 10.1016/j.arth.2023.03.091 PMC10330267

[13] Whittaker, R. K. et al. Variation in taper surface roughness for a single design effects the wear rate in total hip arthroplasty. *J. Orthop. Res.* **35**, 1784–1792 (2017). 27704611 10.1002/jor.23456

[14] Ashkanfar, A., Langton, D. J. & Joyce, T. J. A large taper mismatch is one of the key factors behind high wear rates and failure at the taper junction of total hip replacements: A finite element wear analysis. *J. Mech. Behav. Biomed. Mater.* **69**, 257–266 (2017). 28110182 10.1016/j.jmbbm.2017.01.018

[15] Bormann, T. et al. Influence of FeCl3 and H2O2 in corrosion testing of modular taper connections in total hip arthroplasty: an in vitro study. *Acta Biomater.* **145**, 427–435 (2022). 35417798 10.1016/j.actbio.2022.04.007

[16] Falkenberg, A., Biller, S., Morlock, M. M. & Huber, G. Micromotion at the head-stem taper junction of total hip prostheses is influenced by prosthesis design-, patient- and surgeon-related factors. *J. Biomech.* **98**, 109424 (2020). 31676083 10.1016/j.jbiomech.2019.109424

[17] Wight, C. M., Lanting, B. & Schemitsch, E. H. Evidence based recommendations for reducing head-neck taper connection fretting corrosion in hip replacement prostheses. *Hip Int.* **27**, 523–531 (2017). 29027189 10.5301/hipint.5000545

[18] Racasan, R., Bills, P. J., Blunt, L., Hart, A. & Skinner, J. Method for characterization of material loss from modular head-stem taper surfaces of hip replacement devices. In *Modularity and Tapers in Total Joint Replacement Devices* (eds Greenwald, A. S. , Kurtz, S. M., Lemons, J. E. & Mihalko, W. M.), Vol. STP 1591, 132–146. (ASTM International, 2015).

[19] Brock, T. M. et al. Shorter, rough trunnion surfaces are associated with higher taper wear rates than longer, smooth trunnion surfaces in a contemporary large head metal-on-metal total hip arthroplasty system. *J. Orthop. Res.* **33**, 1868–1874 (2015). 26135357 10.1002/jor.22970

[20] Dransfield, K., Addinall, K. & Bills, P. Comparison and appraisal of techniques for the determination of material loss from tapered orthopaedic surfaces. *Wear* **478–479**, 203903 (2021).

[21] Witt, F. et al. The relation between titanium taper corrosion and cobalt-chromium bearing wear in large-head metal-on-metal total hip prostheses: A retrieval study. *J. Bone Joint Surg. Am.* **96a** (2014). 10.2106/JBJS.M.01199 25232087

[22] Gascoyne, T. C., Turgeon, T. R. & Burnell, C. D. Retrieval analysis of large-head modular metal-on-metal hip replacements of a single design. *J. Arthroplasty* **33**, 1945–1952 (2018). 29402714 10.1016/j.arth.2017.12.044

[23] Martin, A., Van Citters, D., McGrory, B. & Edidin, A. Using coordinate measuring machine validated with white light interferometry to identify contributors to material loss due to corrosion of total hip replacement modular junctions. In *Beyond the Implant: Retrieval Analysis Methods for Implant Surveillance* (eds Mihalko, W. M., Lemons, J. E., Greenwald, A. S. & Kurtz, S. M.), Vol. STP 1606, 118–130. (ASTM International, 2018).

[24] Bishop, N. et al. Wear patterns of taper connections in retrieved large diameter metal-on-metal bearings. *J. Orthop. Res.* **31**, 1116–1122 (2013). 23440943 10.1002/jor.22326

[25] Cook, R. B., Maul, C. & Strickland, A. M. Validation of an optical CMM for the measurement of wear at the taper interface in total hip replacement. In *Modularity and Tapers in Total Joint Replacement Devices* (eds Greenwald, A. S., Kurtz, S. M., Lemons, J. E. & Mihalko, W. M.), Vol. STP 1591, 362–378 (ASTM International, 2015).

[26] Tuke, M., Taylor, A., Roques, A. & Maul, C. 3D linear and volumetric wear measurement on artificial hip joints—Validation of a new methodology. *Precis Eng.* **34**, 777–783 (2010).

[27] Langton, D. J., Sidaginamale, R., Lord, J. K., Nargol, A. V. F. & Joyce, T. J. Taper junction failure in large-diameter metal-on-metal bearings. *Bone Jt. Res.* **1**, 56–63 (2012). 10.1302/2046-3758.14.2000047 PMC3626207 23610672

[28] ASTM F3129-16. Standard Guide for Characterization of Material Loss from Conical Taper Junctions in Total Joint Prostheses 19428 – 2959. (ASTM International, 2016).

[29] Kocagoz, S. B., Underwood, R. J., MacDonald, D. W., Gilbert, J. L. & Kurtz, S. M. Ceramic heads decrease metal release caused by Head-taper fretting and corrosion. *Clin. Orthop. Relat. Res.* **474**, 985–994 (2016). 26847452 10.1007/s11999-015-4683-1 PMC4773353

[30] Arnholt, C. M. et al. Do stem taper microgrooves influence taper corrosion in total hip arthroplasty? A matched cohort retrieval study. *J. Arthroplasty* **32**, 1363–1373 (2017). 28111124 10.1016/j.arth.2016.11.018 PMC5362300

[31] Mueller, U., Braun, S., Schroeder, S., Sonntag, R. & Kretzer, J. P. Same same but different? 12/14 stem and head tapers in total hip arthroplasty. *J. Arthroplasty* **32**, 3191–3199 (2017). 28552447 10.1016/j.arth.2017.04.027

[32] Martin, A. J., Jenkins, D. R. & Van Citters, D. W. Role of corrosion in taper failure and head disassociation in total hip arthroplasty of a single design. *J. Orthop. Res.* **36**, 2996–3003 (2018). 29978908 10.1002/jor.24107

[33] McCarty, C. P., Nazif, M. A., Sangiorgio, S. N., Ebramzadeh, E. & Park, S. H. Can severity of trunnion damage be estimated by visual inspection alone? *Bone Jt. Res.* **12**, 155–164 (2023). 10.1302/2046-3758.123.BJR-2022-0099.R1 PMC10072233 37051817

[34] Morlock, M. M., Dickinson, E. C., Günther, K. P., Bünte, D. & Polster, V. Head taper corrosion causing head bottoming out and consecutive gross stem taper failure in total hip arthroplasty. *J. Arthroplasty* **33**, 3581–3590 (2018). 30100136 10.1016/j.arth.2018.07.017

[35] Kretzer, J. P., Grupp, T. M., Kaddick, C., Mayer, R. T. & Morlock, M. Pre-clinical assessment of the in vivo behaviour of non-articulating interfaces between implant components concerning the consequences of micro motion or corrosion processes: test methodology, and requirements for testing and characterization of implant modularities. In *1st EFORT European Consensus Medical & Scientific Research Requirements for the Clinical Introduction of Artificial Joint Arthroplasty Devices* 104–111 (2023).

[36] Langton, D. J. et al. Material loss at the femoral head taper: A comparison study of the Exeter metal-on-polyethylene and contemporary metal-on-metal total hip arthroplasty. *Bone Joint J.* **100-B**, 1310–1319 (2018). 30295525 10.1302/0301-620X.100B10.BJJ-2017-0406.R3

